# Clinical and cost-effectiveness of a personalised health promotion intervention enabling independence in older people with mild frailty (‘HomeHealth’) compared to treatment as usual: study protocol for a randomised controlled trial

**DOI:** 10.1186/s12877-022-03160-x

**Published:** 2022-06-04

**Authors:** Rachael Frost, Christina Avgerinou, Claire Goodman, Andrew Clegg, Jane Hopkins, Rebecca L. Gould, Benjamin Gardner, Louise Marston, Rachael Hunter, Jill Manthorpe, Claudia Cooper, Dawn A. Skelton, Vari M. Drennan, Pip Logan, Kate Walters

**Affiliations:** 1 Research Department of Primary Care and Population Health, University College London, London, UK; 2 Centre for Research in Public Health and Community Care, University of Hertfordshire, Hatfield, UK; 3 Academic Unit for Ageing and Stroke Research, University of Leeds, Bradford Institute for Health Research, Bradford, UK; 4 Patient and Public Involvement Member, London, UK; 5 Division of Psychiatry, University College London, London, UK; 6 Department of Psychology, King’s College London, London, UK; 7 NIHR Policy Research Unit in Health and Social Care Workforce, King’s College London, London, UK; 8 Department of Physiotherapy and Paramedicine, Glasgow Caledonian University, Glasgow, UK; 9 Centre for Health and Social Care Research, Kingston University & St George’s University, London, UK; 10 Faculty of Medicine & Health Sciences, University of Nottingham, Nottingham, UK

**Keywords:** (3-10) frailty, RCT, Primary care, Ageing, Prevention, Community-dwelling, Behavioural change

## Abstract

**Background:**

Frailty is clinically associated with multiple adverse outcomes, including reduced quality of life and functioning, falls, hospitalisations, moves to long-term care and mortality. Health services commonly focus on the frailest, with highest levels of need. However, evidence suggests that frailty is likely to be more reversible in people who are less frail. Evidence is emerging on what interventions may help prevent or reduce frailty, such as resistance exercises and multi-component interventions, but few interventions are based on behaviour change theory. There is little evidence of cost-effectiveness.

Previously, we co-designed a new behaviour change health promotion intervention (“HomeHealth”) to support people with mild frailty. HomeHealth is delivered by trained voluntary sector support workers over six months who support older people to work on self-identified goals to maintain their independence, such as strength and balance exercises, nutrition, mood and enhancing social engagement. The service was well received in our feasibility randomised controlled trial and showed promising effects upon outcomes.

**Aim:**

To test the clinical and cost-effectiveness of the HomeHealth intervention on maintaining independence in older people with mild frailty in comparison to treatment as usual (TAU).

**Methods:**

Single-blind individually randomised controlled trial comparing the HomeHealth intervention to TAU. We will recruit 386 participants from general practices and the community across three English regions. Participants are included if they are community-dwelling, aged 65 + , with mild frailty according to the Clinical Frailty Scale. Participants will be randomised 1:1 to receive HomeHealth or TAU for 6 months. The primary outcome is independence in activities of daily living (modified Barthel Index) at 12 months. Secondary outcomes include instrumental activities of daily living, quality of life, frailty, wellbeing, psychological distress, loneliness, cognition, capability, falls, carer burden, service use, costs and mortality. Outcomes will be analysed using linear mixed models, controlling for baseline Barthel score and site. A health economic analysis and embedded mixed-methods process evaluation will be conducted.

**Discussion:**

This trial will provide definitive evidence on the effectiveness and cost-effectiveness of a home-based, individualised intervention to maintain independence in older people with mild frailty in comparison to TAU, that could be implemented at scale if effective.

**Trial registration:**

ISRCTN, ISRCTN54268283. Registered 06/04/2020.

**Supplementary Information:**

The online version contains supplementary material available at 10.1186/s12877-022-03160-x.

## Background

Frailty is clinically defined as an accumulation of multiple deficits and a reduction in physiological reserves occurring across multiple body systems as we age, leading to poor recovery from even minor events, such as a urinary tract infection or non-injurious fall [1]. Frailty occurs in approximately 12% of people aged 65 + years worldwide [2] and 14% of older people in England [3]. It is associated with an increased risk of falls, disability, hospitalisation, moves to a care home, dementia, poor quality of life and death [4–7]. Healthcare costs are consistently estimated to be higher in frail older people compared to non-frail older people, largely as a result of increased inpatient costs [8–10]. However, frailty can be understood as a continuum and reversal is possible [11].

Exercise programmes, particularly resistance exercise and in combination with nutrition interventions, can reduce frailty in primary care settings [12]. Current international clinical practice recommendations for frailty support the use of a range of interventions, including: physical activity programmes based on resistance training; social support; development of comprehensive care plans addressing polypharmacy, sarcopenia, treatable causes of weight loss and fatigue; and protein or caloric supplementation if the person has lost weight or has evidence of malnutrition [13]. Pharmacological treatments, vitamin D (unless the person is deficient), psychological therapies or hormone therapy are not currently recommended to treat frailty [13].

Frailty is most commonly conceptualised using the Fried phenotype [14] or Rockwood scale [15]. The Fried criteria groups people experiencing more than three of five possible frailty components as ‘frail’, and those with one or two components as ‘pre-frail’ [14]. Pre-frailty is a risk-state which predisposes an individual to developing frailty [16]. The Rockwood scale classifies people from robust to severely frail, using either cumulative deficits or clinical observation [15]. There is a wide range of differences in functioning across the spectrum of frailty, and little guidance on where to target interventions, although a meta-analysis of 16 studies suggested that over an average of 3.9 years, those who are frail (using the Fried phenotype) are less likely to transition back to robustness than those who are less frail [11]. This suggests targeting earlier stages of frailty is likely to be a more successful approach to preventing decline.

Mild frailty is an intermediate stage on the Rockwood Clinical Frailty Scale where older people experience some loss of physiological reserves but can recover after a stressor event, typically feeling “slowed up”, and requiring greater assistance in instrumental activities of daily living, e.g. cooking, shopping and money management [15]. Around 13% of older people can be classed as ‘mildly frail’ [15]. Mild frailty is associated with adverse outcomes, including a higher risk of death and moving into a care home [15], increased need for care at discharge from hospital [17] and poorer outcomes after post-surgical discharge [18]. Few interventional studies have targeted a mildly frail population with the aim of maintaining independence.

There is little focus in clinical guidelines on frailty prevention, apart from a brief reference to the use of exercise in pre-frailty to prevent frailty [13]. Few UK policies are directed ‘upstream’ to those who are less frail [12], although England’s NHS Long-Term Plan [19] clearly emphasises prevention and ‘supporting people to age well’, including proactively identifying those who would benefit from targeted support to maintain independence. Most studies on frailty prevention (generally targeting those who are pre-frail) have focussed on single or dual intervention domains, typically exercise plus or minus nutrition [20, 21]. Multi-domain interventions including exercise and a range of other domains such as social, nutritional, cognitive training, or medication review show greater promise at reducing or preventing frailty and its related domains across a number of trials [22–25].

However, there is a lack of clarity on which strategies need to be adopted within interventions to maximise effectiveness, as well as absence of a clear theoretical basis, rigorous development process or stakeholder input in intervention development [26]. This can provide challenges when optimising or implementing interventions. Very limited evidence suggests group-based exercise interventions may be more effective than individual sessions, however this may not be feasible to deliver to those who are finding leaving their home more challenging, and there is currently little guidance as to the optimal frequency, intensity, time and type of exercise interventions for frailty management or prevention [13]. More holistic approaches, such as comprehensive geriatric assessment with follow-up visits or preventative home visits, are promising [27, 28] but typically resource-intensive, requiring nurses or a multidisciplinary team, and so present challenges to delivery at scale. The limited number of cost-effectiveness studies available at present showed mixed results. Multidisciplinary team meetings with proactive care management across multiple domains targeted at frail older people, such as The CareWell programme or the Welcheren Integrated Care model, have not shown cost-effectiveness compared to usual primary care [29, 30]. However a resistance training and nutrition education intervention for pre-frail and frail older people with diabetes produced cost savings and increased QALYs [31], whilst a cognitive behavioural intervention to reduce concerns about falls in frail older people was found to be cost-effective [32].

In response to these gaps in the evidence, in an earlier study we developed and feasibility tested an intervention to support independence in older people with mild frailty [33], following Medical Research Council guidelines [34]. The HomeHealth service is a complex theory- and evidence-based behaviour change intervention, arising from an asset-based approach [35], Baltes’ model of ageing [36] and behaviour change theory [37]. Asset-based approaches aim to maximise positive capability and maintain health promoting factors to enhance self-efficacy, problem-solving and coping, in order to retain reserves and functioning [35]. This contrasts to the more commonly used deficit-based approach, which focuses on individuals’ problems and what they lack. Baltes’ model of ageing suggests that successful ageing occurs when an older person prioritises realistic goals or activities that they want to maintain (selection), optimises how these can be performed (optimisation) and adjusts for limitations (compensation) [36]. The behaviour change theory component is based upon the COM-B model, which states that for any behaviour to take place, the person must have sufficient physical and psychological capability, social and physical opportunity and conscious or innate motivation [37]. COM-B provides a framework to link these factors to the specific techniques needed to enable changes in behaviour.

The HomeHealth service is a home-based intervention that we developed based on a series of evidence reviews regarding intervention content and behaviour change techniques used in health promotion interventions for older people [26, 33, 38] and qualitative research with older people with mild frailty, carers and healthcare professionals [39]. It was then co-designed with a range of stakeholders, including older people, healthcare professionals, researchers and voluntary sector representatives [33].

The HomeHealth service was tested in a feasibility randomised controlled trial (RCT) compared to treatment as usual (TAU, usual GP care), in which we recruited 51 older people with mild frailty from four UK general practices, 26 of whom received the intervention. The study successfully recruited within the expected timeframe, with 96% participants remaining in the study at 6 months and minimal missing data (< 1%) [33]. Our process evaluation indicated that the intervention was well-received according to participant interviews and a survey [40], feasible to deliver and had 91% attendance rates for appointments. The service was delivered at modest cost (£307—equivalent $417/patient in Feb 2022). At 6 months we found significantly better functioning (Barthel Index; + 1.68, p = 0.004) and grip strength (+ 6.48 kg, p = 0.02), reduced psychological distress (GHQ-12; -3.92, p = 0.01) and increased capability-adjusted life years (+ 0.017, p = 0.03) in the intervention arm compared to treatment as usual. There were no differences in other outcomes. Given the promising nature of the intervention, we aim to test the effectiveness and cost-effectiveness of HomeHealth in a definitive trial across a wider range of participants, providers and contexts.

### Aims

This trial aims to:

1. Test the clinical effectiveness of HomeHealth in maintaining independence in a RCT in comparison to TAU.
2. Determine the cost-effectiveness of HomeHealth in comparison to TAU.
3. Quantify the costs and savings of HomeHealth to different health and social care providers.
4. Explore the context, mechanisms and impact of the intervention for different populations (age, gender, deprivation, ethnicity, rurality) and barriers and facilitators to implementation at scale.

## Methods

We will carry out a two-arm, single-blind, parallel-group RCT comparing HomeHealth to TAU, including a cost-effectiveness analysis and mixed methods process evaluation.

### Eligibility criteria

We will include community-dwelling older people (including those in sheltered or extra care housing – no care workers on site) aged 65 + who are registered with a general practice in the participating site area; scoring as ‘mildly frail’ on the Clinical Frailty Scale (CFS score of 5), which is defined as ‘more evident slowing, who need help or support in higher order instrumental activities of daily living (e.g. finances, heavy housework), with progressive impairment of outdoor mobility, shopping and housework’ [15]; life expectancy of > 6 months; and capacity to consent to participate.

We will exclude care home residents; people with moderate-severe frailty (CFS score of 6–9) or not frail (CFS score of 1–4); receiving palliative care; or already case managed (e.g. receiving a similar ongoing intervention from the voluntary sector or a community matron).

### Intervention

A detailed breakdown of the HomeHealth service is provided according to the TIDIER checklist [41] in supplementary file 1. HomeHealth is delivered over approximately six appointments in the older person’s home. Three appointments is considered a minimum dose, whilst participants could receive up to a maximum of 12 if they have particularly complex needs. These were originally planned to be delivered face-to-face with some telephone interim support, however they were adapted to be delivered by phone or video call, as needed, in light of the Covid-19 pandemic. If needed, internet-enabled tablets can be offered to participants receiving the intervention remotely to facilitate communication.

HomeHealth follows an intervention manual. In the first appointment, a HomeHealth worker comprehensively assesses the person, with a particular focus on socialising, mobility, nutrition and psychological wellbeing (including mood and memory), but including any other relevant issues (e.g. pain, continence, caring). Participants are encouraged to identify an outcome goal for the service that is important for them, and at either the first or second appointment are encouraged to break this down into behavioural goals, which are then operationalised into SMART (specific, measurable, achievable, relevant, timely) goals that are achievable in small steps over the course of the intervention. Goals can include a range of activities, such as home-based exercises based on the Otago programme (which shows effectiveness at reducing falls [42] and improving balance [43]), identifying social activities to attend or making dietary changes to improve protein or calorie intake. The COM-B model [37] is used by the HomeHealth worker to assess capability, opportunity and motivation to achieve the behavioural goal and to identify any barriers, developing an action plan with the person as to how these might be overcome. These are documented on a Health and Wellbeing Plan, and self-monitoring forms (e.g. diaries) may be used to record completion of activities such as exercises. Where relevant, equipment (e.g. weights or resistance bands) or information resources (e.g. details of local social groups) is provided to the older person.

At each subsequent appointment, progress towards achieving goals is reviewed, with changes made as needed to resolve any issues or to extend goals (e.g. increasing repetitions or resistance in strength exercises). An emphasis is placed on how new behaviours could be maintained over time, and habit formation. Further goals may be identified over following sessions. There are no restrictions on accessing other services or treatments during the study period.

HomeHealth is delivered by trained HomeHealth workers, who are not required to have a specific health or social care qualification but to have some experience in working with older people (for example, in the voluntary or not for profit sector). HomeHealth workers are based in local voluntary sector services in each area and are supervised by a centrally located Team Leader, in biweekly remote group supervisions with one-to-one support as needed. HomeHealth training is delivered entirely remotely as a blend of live and asynchronous content delivered by specialists in that area, including pre-recorded webinars and live seminars, activities and a strong emphasis on case-based discussions. The training time is intended to be equivalent to a one-week course. HomeHealth workers also have access to remote support from the expert course providers (in behaviour change, exercise for older people, nutrition and psychological wellbeing) via email for specific queries on tailoring the intervention for individual clients.

The control arm will receive TAU. This is defined as standard care that any eligible patient aged 65 + would normally receive in England in primary care (e.g. contact with the GP or the practice nurse), including any other community or secondary care input as a result of referral by a GP. No mild frailty-specific interventions are currently widely available in the UK.

### Setting and recruitment

Participants will be recruited from general practices in England, in one of three areas (North Thames Region, East & North Hertfordshire and West Yorkshire). Practices will be asked to undertake list searches to identify those fitting the eligibility criteria above, using the frailty tool available to the practice (usually the Electronic Frailty Index) [44], which will then be screened by clinicians to remove known ineligible patients. Practices will then send invitation letters, study leaflets and reply slips to potentially eligible patients. Potential participants can also be referred to the study directly by clinicians in participating General Practices or local voluntary sector staff. We will undertake community-based recruitment such as presenting to local groups and providing leaflets to relevant community organisations, targeting under-served populations to maximise diversity of our sample.

Researchers will telephone screen potential participants according to the inclusion criteria, and if eligible, will post an information sheet and invite them to an appointment (face-to-face or remote) to seek consent and undertake a baseline assessment. An interpretation service is available if required. After completion of a baseline assessment and confirmation of eligibility, participants will be randomised.

### Randomisation

We will randomise participants 1:1 (stratified by site) to receive the HomeHealth service or TAU. Randomisation will be carried out by unblinded staff members using the remote computerised web-based application ‘Sealed Envelope’, provided by UCL's Priment Clinical Trials Unit. Outcome assessors, the Chief Investigator, the Trial Manager and Trial Management Group members who are not site Principal Investigators or responsible for intervention delivery will be blinded to participant allocation.

### Outcomes

Clinical outcomes will be measured at baseline, 6 months and 12 months by a researcher blind to intervention status (see Table 1). Maintenance of blinding will be documented using a Researcher Perception form. Assessments will be completed face-to-face at the participant’s home or remotely (by video or telephone), according to any government guidelines for prevention of Covid-19 infection and the participant’s preferences. Assessments can be divided into two sessions if needed. Participants receive a £10 voucher (max 3) for completing each assessment. Data will be kept confidentially at sites and entered in a secure web-based database developed for the trial. A monitoring plan is in place to ensure data quality.

**Table 1:** List of data collected and timepoints

Construct	Measure used (supporting references)	Baseline	6 months	12 months	Anytime	Extracted from medical notes
Independence in ADLs	Modified Barthel Index [45]	✓	✓	✓		
Instrumental ADLs	Nottingham Extended Activities of Daily Living [46]	✓	✓	✓		
Frailty	Fried Frailty Phenotype score [14]	✓	✓	✓		
a.Gait speed	Self-reported according to Op het Vald’s (2018) questionnaire [47]	✓	✓	✓		
b. Grip strength	Self-reported according to Op het Vald’s (2018) questionnaire [47]	✓	✓	✓		
c. Physical activity	International Physical Activity Questionnaire-Elderly [48], quantified according to the E guidelines [49]	✓	✓	✓		
d. Exhaustion	Exhaustion questions from 7-item Centre for Epidemiological Studies Depression Scale “7. I felt that everything I did was an effort,” “20. I could not get going.” [14]	✓	✓	✓		
e. Weight loss	Weight loss question from the Mini-Nutritional Assessment Short Form [50]	✓	✓	✓		
Quality of life and Quality-adjusted Life Years	EuroQol-5D-5L [51]	✓	✓	✓		
Capability and Capability-adjusted Life Years	ICEpop CAPability measure for Older people (ICECAP-O) [52]	✓	✓	✓		
Wellbeing	Warwick-Edinburgh Mental Wellbeing Scale (WEMWBS) [53]	✓	✓	✓		
Psychological distress	General Health Questionnaire-12 [54]	✓	✓	✓		
Loneliness	University of California, Los Angeles 3-item loneliness scale [55]	✓	✓	✓		
Cognition	Montreal Cognitive Assessment (MoCA) [56] or telephone MoCA (remote items only) [57, 58]	✓	✓	✓		
Falls	ProFANE consensus definition [59]	✓	✓	✓		
Mortality	Medical notes or family report		✓	✓	✓	
Carer burden	Adapted from adapted iMTA Valuation of Informal Care Questionnaire [60]	✓	✓	✓		
Demographics	Questionnaire developed for trial	✓				
Alcohol intake	Alcohol Use Disorders Identification Test – consumption (AUDIT-C [61])	✓				
Deprivation level	Local area Index of Multiple Deprivation based on postcode [62]	✓				
Smoking status	Single question	✓				
Covid-19 status	Questions on infection status and ongoing symptoms	✓	✓	✓		
Unpaid care and use of primary and community health and social care services	Modified Client Services Receipt Inventory [63]	✓	✓	✓		
Use of health services, medications and number of long-term conditions						✓
Adverse events			✓	✓	✓	✓

The primary outcome is the Modified Barthel Index (BI) [45] measured at 12 months, which is interviewer-administered with scoring based on discussions with participants. The BI measures an individual’s ability to undertake basic Activities of Daily Living (ADLs) and is widely used, correlates well with need for home care [64] and is considered a key outcome measure in frailty trials [65]. It measures what people actually do as opposed to what they could do. Our intervention arm showed significantly higher scores than usual care in our feasibility RCT at 6 months [33]. Our secondary outcomes and variables collected at each timepoint are reported in Table 1.

The study was planned before Covid-19 (winter 2019) and we intended to conduct face-to-face research assessments, measuring frailty using handgrip strength (dynamometer), gait speed (time taken to walk a measured distance) and weight loss (measured using scales). Given the restrictions associated with the pandemic that were implemented in March 2020, we revised our protocol to use self-report measures (validated in older people across a range of frailty levels [47]) to enable remote assessments. When research assessments are conducted face-to-face, we will additionally carry out physical gait speed assessment (m/s) [66] and grip strength assessment using a dynamometer (kg, highest score out of three trials) [67] to confirm the validity of the self-report measures in our population with mild frailty.

### Safety

Researchers will collect Adverse Events (AEs) and Serious Adverse Events (SAEs) at 6-and 12-month timepoints, and participants will be encouraged to report any AEs or SAEs experienced between assessments by contacting researchers. HomeHealth workers (HHWs) will also report adverse events. All AEs and SAEs will be reviewed by a site Principal Investigator and/or Clinical Safety Lead (CSL), and all SAEs will be reviewed by the Chief Investigator. Only SAEs related to the intervention will be reported to the Sponsor. Researchers will not have access to relatedness assessments in order to maintain blinding. Relatedness to the intervention will be assessed by the site Principal Investigator and/or CSL in a blinded manner for AEs and SAEs reported by researchers, and unblinded for AEs and SAEs reported by HHWs. The reporting of SAEs will be monitored by Priment CTU. Pharmacovigilance standard operating procedures will be followed.

### Health economic evaluation

Healthcare resource use (contacts with primary, community and secondary care, hospitalisations, procedures and medications) will be extracted from patient medical records. Service resource use not available from files and wider services (e.g. of voluntary sector and social care) will be collected using a modified Client Services Receipt Inventory (CSRI) [63] at 0, 6 and 12 months, asking about the previous 6-months. The CSRI has been modified based on the population in our feasibility study to include the range of services they may use (e.g. podiatry, opticians, hearing aids, dental care, physiotherapy, exercise classes, day care, etc.). Health and social care resource use will be costed using nationally published sources (PSSRU [68], NHS Reference Costs [69] and the British National Formulary [70]). We will additionally ask about unpaid and paid (state and out-of-pocket) carer time for specific activities of daily living, using an adapted iMTA Valuation of Informal Care Questionnaire (iVICQ) [60]. This will be costed as the cost of face-to-face local authority-funded homecare worker time to reflect the fact that if this care were to be reduced homecare workers would be the alternative to unpaid carers providing this assistance.

Intervention costs (including staff training, administration, supervision and delivery) will be included in the costs of the intervention group.

### Process evaluation

The process evaluation will collect quantitative and qualitative data alongside the main trial. Feedback on service delivery (e.g. fidelity) will not be given to service providers or other team members until after trial completion. An independent researcher will carry out up to 40 semi-structured audio-recorded interviews with participants receiving the service, service providers and other stakeholders, using topic guides developed with our public and patient involvement and engagement (PPIE) representatives and stakeholders from our implementation group. We will additionally explore the impact of remotely delivering the service in the time of Covid-19 and its influence upon participant and provider experiences.

HomeHealth workers will keep an ongoing record of service delivery process data, including number of appointments attended, modality, technical issues experienced if delivering remotely, duration and reasons for non-attendance. Goals will also be recorded, and will be coded into mobility, psychological, social, memory, nutrition or other. We will ask providers to rate progress towards achieving goals, including 0–2 ratings for each SMART goal (0 = no progress, 1 partially met/some progress, 2 = met/exceeded goal) and goal attainment scaling for outcome goals (from -2 (much less than expected progress) to + 2 (much more than expected progress) [71]). Fidelity will be assessed through fidelity checklists, developed for this intervention and based on what should be included at each appointment. These will be completed after each appointment by HomeHealth workers. We will record all audio appointments, and independently verify fidelity using the same checklist by a trained rater, who will check recordings for all appointments from 10% individuals receiving the intervention.

We will also utilise trial data to explore mechanisms of impact, including demographic data to assess reach and outcome data to assess whether setting goals on one topic will affect the hypothesised linked outcome (e.g. psychological goal and psychological distress scores).

### Sample size

We are aiming to recruit a sample size of 386 people (193 per arm). This will provide 90% power at the 5% significance level to detect a minimum clinically important difference of 1.85-points [72] in the BI, with a standard deviation (SD) of 5. We found a SD of 3 in the feasibility RCT, however in frail populations larger SDs have been reported [20, 22] so we have conservatively assumed a larger SD. Using 90% power and 5% significance level, this would require 308 people (154 per group). Attrition was minimal (6%) over 6 months in our feasibility study, however other studies have reported higher attrition rates with longer follow-up [8] so we have conservatively assumed 20% attrition over 12 months. The first six months will form a pilot phase based on site-specific and overall recruitment rates (target red < 80, amber 80–159, green 160 +), with measures put in place if we are not recruiting at ‘green’.

We have not adjusted for clustering by therapist (HomeHealth worker) as we anticipated that this will be minimal and non-significant. No trials in those with mild frailty have reported intraclass correlation coefficients (ICCs) for therapist clustering, only clustering by GP practice in cluster RCTs in older community-based general populations [73]. Unpublished PhD thesis data examining therapist effects in a secondary analysis of a cluster exercise trial in older people [74] did not find significant clustering by therapist (ICC 0.01, *P* = 0.54).

### Participant timelines

Participants will be involved in the RCT for 12 months. Participants will be randomised within four weeks of completing the baseline assessment to HomeHealth or TAU and 6 months (i.e. end of intervention) and 12 months after randomisation all participants will be invited to undertake outcome assessments. With participants’ consent, data on healthcare usage, long term conditions and medications will also be extracted from their medical notes. Approximately 20 intervention arm participants will also be asked to take part in a process evaluation interview after their 12-month assessment.

### Statistical methods

A detailed statistical analysis plan, including the health economic analysis, will be developed a priori and reviewed and approved by the Trial Steering Committee. All analyses will be by intention to treat. As missing data in the feasibility study were low and there are few gains to multiple imputation in RCTs [75, 76], imputation will not be used. We will descriptively summarise participants’ baseline characteristics using appropriate summary statistics (mean and SD, median and interquartile range or proportions) by randomised group.

The primary outcome (BI score at 12 months) will be analysed using linear mixed models, including 6 and 12-month data, controlling for baseline BI score and site (the stratification variable). Assumptions will be checked and appropriate transformations or analogous models will be used if the assumptions of linear models are violated. The ICC will be reported. Continuous secondary outcomes will be analysed using similar linear mixed models to the primary outcome, including 6 and 12-month data and controlling for outcome baseline score and site. Dichotomous outcomes (death and exhaustion) will be analysed using logistic regression. Falls will be analysed using Poisson regression or an analogous alternative if the assumptions for Poisson are not met. If it is not possible to use Poisson regression or an analogous alternative, then we will consider dichotomising falls and analysing in a similar way to other dichotomous outcomes. If there are too few events to perform statistical modelling for falls or the other dichotomous outcomes, they will be reported descriptively.

We will examine baseline predictors of missingness for the primary outcome and include any significant predictors of missingness in a supportive analysis to restore the missing at random assumption using a similar model to the primary analysis. All analyses will be complete case. We will also perform a complier average causal effects (CACE) analysis after unblinding using a threshold dosage of 3 + sessions for compliance to determine the average treatment effect of participants who would have adhered to the protocol regardless of how they were randomised.

For the process evaluation, we will carry out a number of exploratory analyses, including whether those who get a therapeutic dose of HomeHealth (3 + sessions) have higher BI at 12 months; whether choice of goal impacts on related outcomes (e.g. social goal and UCLA-3 score) and whether progress towards goals is associated with higher BI scores. If sufficient data are available, we will explore the impact of remote delivery vs. face to face delivery on outcomes. We will descriptively summarise service delivery data (e.g. appointments attended) and compare the demographics of those recruited to the expected population to assess intervention reach. Qualitative data will be analysed using codebook thematic analysis [77], involving multiple multidisciplinary team members (including Patient and Public Involvement and Engagement (PPIE)), and barriers and facilitators to implementation will be explored using Normalisation Process Theory [78].

For the health economic analysis, we will calculate the mean incremental cost per quality adjusted life years gained (QALYs) using EQ-5D-5L and the relevant UK tariff. QALYs will be calculated from the EQ-5D-5L as the area under the curve adjusting for baseline [74] with site as a fixed effect in line with the analysis of the primary outcome. We will also report Years of Full Capability (YFC) using ICECAP-O and its respective tariff for the duration of the trial. YFC will be calculated according to the most current guidance [79]. The primary analysis will be the incremental cost per QALY gained from a health and personal social services cost perspective, with secondary analyses reporting the incremental cost per YFC gained, and calculating incremental costs from a wider cost perspective to capture the impact on carers and any patient/carer out of pocket costs for health and social care. Means and 95% confidence intervals will be based on bootstrapped results. We will adjust difference in total cost at 12 months by baseline values [80] with site as a fixed effect and a random effect for therapist clustering.

EQ-5D-5L, ICECAP-O, QALYs and YFC, resource use and costs will be summarised descriptively. Cost-effectiveness acceptability curves and cost-effectiveness planes will be reported to represent the probability that the intervention is cost-effective compared to TAU for a range of cost-effectiveness thresholds for a QALY and YFC gained. Seemingly unrelated regression will be used to account for the correlation between costs and QALYs/YFC. A range of sensitivity analyses will be conducted for any assumptions. Procedures for handling missing data will follow those of the statistical analysis.

We will carry out a budget impact analysis, developing a tool for funders or commissioners to use to assess the yearly costs to their budget of implementing HomeHealth in a range of different commissioning models. Due to the paucity of long-term data upon which to base assumptions regarding effectiveness over time, we will explore alternative scenarios, including: 1) assuming constant effectiveness of the intervention over five years, 2) initial further gains in year 1–2 (if care home moves and hospital admissions are avoided) followed by a depreciation in effect, and 3) a slow decline in effectiveness over time. We will also include the impact of further assumptions such as the grade of intervention delivery staff, training costs and patient case load, taking into account the size and composition of the relevant local population. We will model scenarios for potential costs to the NHS if aspects of the intervention were commissioned by a third sector organisation, or if commissioned as part of publicly funded social care, the impact on local authorities.

### Patient and public involvement and engagement (PPIE)

PPIE members are involved at all stages of the project. PPIE is led by JH (PPIE lead) and RF (academic lead for PPIE), who maintain regular contact regarding the trial and PPIE activity. Three PPIE members, two of whom were involved in the earlier feasibility study, were involved in developing the grant application, and they will join an Implementation Group that will look at service delivery and how to facilitate implementation throughout the trial and in future (if effective). JH attends all Trial Management Group meetings and two other PPIE members are invited to attend when of interest. JH reviewed the Participant Information Sheet, consent form and study leaflets prior to ethics submission. Three further independent PPIE members also sit on the Trial Steering Committee.

JH, who is blind, and another PPIE member, who is hearing-impaired, have also helped research assistants to pilot remote consent and baseline assessments over phone or zoom in order to optimise the research process. With regards to training HomeHealth workers, two PPIE members drafted a section of the intervention manual regarding remote communication with older people, and JH was involved in a remote exercise training demonstration video.

PPIE members have repeatedly raised the issue of the difficulties of under-represented groups participating in the study such as those with limited English or who lack digital confidence or the necessary equipment or broadband connection. Aiming to address this problem, the study will provide internet enabled tablets to participants receiving the intervention, if needed, to enable better communication, particularly for aspects of the intervention such as exercise. Language is not an exclusion criteria for the trial, and a translation service has been set up for use in screening, baseline assessments and intervention delivery, and bilingual staff members at sites will provide interpreting support if needed.

### Oversight & monitoring

HomeHealth RCT has an independent Trial Steering Committee and independent Data Monitoring and Ethics Committee (DMEC). Both committees will meet twice yearly to review the trial and make recommendations. The DMEC will review all safety data from the trial. There are no planned interim analyses.

### Ethics

The study has been approved by the Health Research Authority Social Care Research Ethics Committee (ref 20/IEC08/0013). Any amendments will be approved by the sponsor and communicated to all sites and the Health Research Authority. Researchers will seek audio recorded verbal consent (if remote) or written consent (if face-to-face) from all participants after being sent an information sheet and given the opportunity to ask questions to the researcher. Researchers will be trained in Good Clinical Practice and the Mental Capacity Act 2005. If a participant loses capacity during the study, we will retain them in the study if they have consented to this at baseline and if a personal or nominated consultee can be identified to advise on whether they would likely want to continue and to support them with assessments. Any concerns about participants’ welfare will be discussed with KW or CA (practising GPs) or AC (practising geriatrician) or intervention supervisors and appropriate local services informed with the participant’s consent where possible.

### Dissemination

Trial results will be disseminated through academic publications and conferences in the fields of geriatrics, gerontology and primary care. We will present findings in appropriate local forums for health and social care professionals, voluntary sector services and local interest and service user groups. The study results will also be made available in an accessible format using age appropriate and user-friendly language. Participants who have indicated they are interested in the results will be sent a summary of the findings. The trial is registered in the ISRCTN database, which will be updated as appropriate.

## Discussion

This RCT will evaluate the effectiveness of an intervention focused on behaviour change for maintaining independence in older people with mild frailty at 12 months. This intervention provides a novel approach due its inclusion of multiple domains (mobility, nutrition, psychological and social); focus on behaviour change and maintenance; and theoretical basis and rigorous development process, including co-production workshops with a wide range of stakeholders and public involvement. HomeHealth is also delivered by the voluntary sector using trained, non-specialist support workers. In the UK, the voluntary sector typically delivers related services such as care navigation and social prescribing services, indicating this is likely to be a scalable and sustainable approach. In current practice, exercise and nutrition services are usually provided separately and may not be tailored to those who are becoming frail. HomeHealth provides a more holistic approach targeted at a specific population in order to prevent decline, and has been adapted for any Covid-19 restrictions and more remote delivery if needed.

This trial will provide important evidence regarding the effectiveness and cost-effectiveness of HomeHealth. Our primary outcome is independence as measured by the Barthel Index, a key outcome for older people that measures actual activity [65]. Frailty trials are increasingly using the Fried phenotype as an outcome, however, currently this lacks validation, it is restricted by a score of 0–5 for components of frailty and its meaningfulness to older people is unknown. Loss of independence in activities of daily living indicates a need for care and support, so as well as being meaningful for older people, this will also provide important information for health and social care.

HomeHealth targets a population early in the frailty trajectory, with a relatively low resource intervention. This has the potential to reduce the demand on health and social care, which we will explore through a health economic analysis, including a budget impact analysis across health and social care sectors. This will provide vital evidence for commissioners’ decision making. If effective and cost-effective, HomeHealth is a manualised intervention with an established training package and supervision structure that could quickly be commissioned, scaled up and implemented in UK and other similar primary care/voluntary sector settings. Our process evaluation, which will explore equity of access, context and mechanisms, will facilitate planning of service implementation and adaptation to local contexts. We have also formed an implementation group including providers and key stakeholders, who will meet throughout the trial to make recommendations about how HomeHealth can best be implemented more widely.

### Limitations

As HomeHealth is a behaviour change intervention, participants cannot be blinded to arm allocation, which may bias participant reported outcomes. Researchers carrying out assessments and key trial management staff will be blinded to arm allocation to reduce the risk of bias. Systems and strategies to maintain blinding will be put in place (e.g. remote outcome assessments with an alternative blinded researcher from another site, where a researcher becomes accidentally unblinded during the course of the study).

HomeHealth showed promise of effectiveness in the initial feasibility study [33]. However, it should be noted that a number of adaptations were needed for trial processes and the intervention in light of the Covid-19 pandemic, including making provisions for remote intervention delivery and remote assessments. The context of the trial also changed due to the pandemic (e.g. TAU, including access to routine services, and levels of social isolation have changed). Our process evaluation will explore the impact of this, in order to inform future recommendations.

A larger number of intervention providers will be delivering the HomeHealth service than in the feasibility study, which may affect consistency across participants or sites. In order to reduce the risk of drift, HomeHealth workers will have regular case-based supervision from a centrally located Team Leader and we will calculate ICCs to explore therapist effects. Fidelity of delivery will also be explored in the process evaluation.

## Supplementary Information

**Additional file 1:** **Supplementary Table 1. **TIDIER checklist forHomeHealth

## Data Availability

The datasets generated and/or analysed during the current study during this study will be included in the subsequent results publication. Access to the quantitative datasets generated and/or analysed during the current study will be included in the subsequent results publication, where they can be sufficiently de-identified for data-sharing and conform to ethics and data governance requirements. The primary qualitative data will not be shared as it is not possible to de-identify this data sufficiently and retain the integrity of the data.

## Abbreviations

ADL: Activities of daily living
AE: Adverse Event
BI: Barthel Index
CFS: Clinical Frailty Scale
COM-B: Capability, Opportunity, Motivation and Behaviour
CSRI: Client Services Receipt Inventory
CSL: Clinical Safety Lead
DMEC: Data Monitoring and Ethics Committee
EQ-5D-5L: Euro-Qol-5D-5L
GHQ-12: 12 Item General Health Questionnaire
GP: General Practitioner
ICC: Intraclass Correlation Coefficient
ICECAP-O: ICEpop CAPability measure for Older people
ISRCTN: International Standard Randomised Controlled Trial Number
MoCA: Montreal Cognitive Assessment
NHS: National Health Service
PPIE: Public and Patient Involvement and Engagement
QALY: Quality-adjusted Life Year
RCT: Randomised Controlled Trial
SAE: Serious Adverse Event
SD: Standard deviation
TAU: Treatment as Usual
t-MoCA: Telephone Montreal Cognitive Assessment
UCL: University College London
UK: United Kingdom
WEMWBS: Warwick-Edinburgh Mental Wellbeing Scale
YFC: Years of Full Capacity
