# Supplementary material for: Clinical and cost-effectiveness of a personalised health promotion intervention enabling independence in older people with mild frailty (‘HomeHealth’) compared to treatment as usual: study protocol for a randomised controlled trial
Source: BMC Geriatr. 2022 Jun 4;22:485. doi: 10.1186/s12877-022-03160-x (PMC9166243; doi:10.1186/s12877-022-03160-x)
Supplement: Supplementary file 1 — Additional file 1: Supplementary Table 1. TIDIER checklist forHomeHealth [file 12877_2022_3160_MOESM1_ESM.docx]

**Supplementary Table 1. TIDIER checklist for HomeHealth**

| **TIDIER item** | **Application in HomeHealth** |
| --- | --- |
| 1 Brief name: Provide the name or a phrase that describes the intervention | HomeHealth |
| 2 Why: Describe any rationale, theory, or goal of the elements essential to the intervention | An asset-based approach underpins the overall intervention approach, focussing on maintaining independence, health and current activities rather than addressing deficits.  Baltes’ theory of successful ageing (selection, optimisation and compensation) informs the overall intervention approach and choice of goals.  The COM-B model (Capability, Opportunity Motivation – Behaviour) is used during the goal setting process to identify barriers to behaviour change and build strategies into the action plan to overcome these barriers. |
| 3 What materials: Describe any physical or informational materials used in the intervention, including those provided to participants or used in intervention delivery or in training of intervention providers. Provide information on where the materials can be accessed (such as online appendix, URL) | - HomeHealth intervention manual for provider training - Training materials include a combination of self-directed pre-recorded lectures and activities and structured live sessions, on the topics of communication skills, behaviour change, nutrition, exercises and psychological wellbeing. - Resource packs (educational information for the service providers, local services to signpost to and resources to hand out to clients e.g. leaflets) in the four domains of mobility, nutrition, psychological wellbeing and socialising, but also for common related issues such as pain or continence. Existing national resources are provided by topic experts, supplemented with local resources added over time as service delivery progresses. - Equipment, mainly adjustable ankle weights, grip strengtheners (of varying resistance) and resistance bands (of varying resistance), are supplied to clients if they have set a relevant exercise goal. - Forms to be completed with the client, including health and wellbeing plans (which provides a format for goal setting and action planning) and client notes. Self-monitoring forms are given to the client to complete if relevant to the goal (e.g. an exercise diary). - Phone or video calling is required for remote delivery. Internet-enabled tablets may also be provided to participants with no access to video calling. - Personal protective equipment is worn in accordance with relevant guidelines in relation to Covid-19 at the time. |
| 4 Procedures: Describe each of the procedures, activities, and/or processes used in the intervention, including any enabling or support activities | First appointment (1-2hrs): building rapport with the older person; understanding their current situation with regards to the four main domains of HomeHealth (mobility, nutrition/diet, socialising and psychological wellbeing); identifying an outcome goal to work on; setting an action before the next appointment. If an outcome goal is readily selected, this appointment can also include breaking the outcome goal down into a behavioural then SMART goal; assessing potential barriers using COM-B; developing an action plan incorporating ways to overcome any barriers; and writing this in the health and wellbeing plan, along with how progress will be recorded.  Second appointment (30-60min): If a SMART goal was not set at the end of the first appointment, this appointment includes SMART goal setting as described above. If a goal was set, this appointment focuses on reviewing progress towards goals, providing feedback, forming habits, modifying goals as needed to overcome barriers or selecting new goals. If needed, the HomeHealth workers explore ways to cope with setbacks or build motivation. HomeHealth workers may provide information, signposting or equipment as needed.  Subsequent appointments (3-5, 30-60min): Reviewing progress towards goals with modifications, equipment or signposting as needed, as described above.  Final appointment (6, 30min): reinforce self-efficacy, remind the participant of new things they have learned and achieved, advice and planning for maintaining motivation after HomeHealth and forming habits, information on further help and support.  Contact may be made between appointments to provide information or confirm appointments, but this is expected to be limited.  Behaviour change functions include education, training, enablement and environmental restructuring (e.g. helping the person to access home modifications or assistive equipment to enable them to continue activities or reach a goal). Behaviour change techniques (BCTs) include goal setting, action planning and problem solving, reviewing progress, providing feedback, with other BCTs (e.g. encouraging self-monitoring) used where applicable. |
| 5 Who provided: For each category of intervention provider (such as psychologist, nursing assistant), describe their expertise, background, and any specific training given | HomeHealth workers: non-specialist support workers with experience in working with older adults, who are specifically trained for this role. Typically they may have a background working in the voluntary sector or social prescribing, but are not required to have a particular qualification. They receive a training package equivalent to one week full time training, including communication skills, behaviour change, nutrition, psychological wellbeing and strength and balance exercises (based on the Otago home-exercises model). This includes a mix of pre-recorded and live sessions. They receive biweekly group supervision by a Team Leader and one-to-one support as needed. They have access to topic experts for specialist queries (e.g. exercise progression). |
| 6 How: Describe the modes of delivery (such as face to face or by some other mechanism, such as internet or telephone) of the intervention and whether or not it was provided individually or in a group | Face-to-face individual appointments (with PPE as needed according to government guidelines) and/or video calling or telephone appointments. A carer may also be present where the person wishes or if they have significant cognitive impairment. Project workers also liaise with GPs, therapists or other services as needed. |
| 7 Where: Describe the type(s) of location(s) where the intervention occurred, including any necessary infrastructure or relevant features | Participants’ homes, requiring time and budget for travel and secure storage of notes and health and wellbeing plans during transit. If delivered remotely, participants require a phone or internet-enabled device. |
| 8 When and how much: Describe the number of times the intervention was delivered and over what period of time including the number of sessions, their schedule, and their duration, intensity or dose | Planned as six sessions over a six month period, with some flexibility as to the timing and amount. Three appointments is considered a minimum dosage, and if they have very complex needs a maximum of 12 appointments can take place. The first appointment is approximately 1-2 hours and subsequent appointments last around 30 min (max 60min). |
| 9 Tailoring: If the intervention was planned to be personalised, titrated or adapted, describe what, why, when, and how | The intervention is tailored according to the needs and goals of the participant. Although based around the 4 key domains of mobility, nutrition, psychological wellbeing and socialising, the outcome goals, behavioural goals and SMART goals are developed with the individual. Behaviour change techniques selected are also tailored, with COM-B used to identify individual potential barriers to overcome and these are built into the action plan. The total number and duration of appointments follows the guide outlined above but can be tailored within this guide as needed. |
| 10 Changes: If the intervention was modified during the course of the study, describe the changes (what, why, when, and how) | n/a |
| 11 How well – planned: If intervention adherence or fidelity was assessed, describe how and by whom, and if any strategies were used to maintain or improve fidelity, describe them | Fidelity: self-reported provider checklists, plus independent rating of audio recorded appointments with 10% of those receiving the intervention. No feedback is given to HomeHealth workers.  Progress towards goals: Progress towards outcome goals is documented using Goal Attainment Scaling, progress towards SMART goals is documented on a provider-rated 0-2 scale (0=not met to 2=fully met/exceeded).  Appointments attended, duration and mode of delivery is recorded for each participant. |
| 12 How well – actual: If intervention adherence or fidelity was assessed, describe the extent to which the intervention was delivered as planned | n/a |
